# Spontaneous shrinking of soft nanoparticles boosts their diffusion in confined media

**DOI:** 10.1038/s41467-019-12246-x

**Published:** 2019-09-20

**Authors:** Pierre-Luc Latreille, Vahid Adibnia, Antone Nour, Jean-Michel Rabanel, Augustine Lalloz, Jochen Arlt, Wilson C. K. Poon, Patrice Hildgen, Vincent A. Martinez, Xavier Banquy

**Affiliations:** 1 Faculty of Pharmacy, Université de Montréal, PO Box 6128, Succursale Centre-ville, Montréal, QC H3C 3J7 Canada; 2 School of Physics and Astronomy, The University of Edinburgh, Peter Guthrie Tait Road, Edinburgh, EH9 3FD UK; 3 Present Address: Research Institute of the McGill University Health Centre, Injury Repair Recovery Program, Department of Surgery, Division of Orthopaedics, Montreal, QC Canada; 4 Present Address: INRS-Institut Armand-Frappier Research Centre, 531, boul. des Prairies, Laval, QC H7V 1B7 Canada

**Keywords:** Colloids, Gels and hydrogels, Nanoparticles

## Abstract

Improving nanoparticles (NPs) transport across biological barriers is a significant challenge that could be addressed through understanding NPs diffusion in dense and confined media. Here, we report the ability of soft NPs to shrink in confined environments, therefore boosting their diffusion compared to hard, non-deformable particles. We demonstrate this behavior by embedding microgel NPs in agarose gels. The origin of the shrinking appears to be related to the overlap of the electrostatic double layers (EDL) surrounding the NPs and the agarose fibres. Indeed, it is shown that screening the EDL interactions, by increasing the ionic strength of the medium, prevents the soft particle shrinkage. The shrunken NPs diffuse up to 2 orders of magnitude faster in agarose gel than their hard NP counterparts. These findings provide valuable insights on the role of long range interactions on soft NPs dynamics in crowded environments, and help rationalize the design of more efficient NP-based transport systems.

## Abstract (web summary)

Understanding the transport of nanomaterials, especially nanoparticles, through a porous medium has major implications in many different fields. Here the authors report the ability of soft nanoparticles to shrink in confined environments, therefore boosting their diffusion compared to hard, non-deformable particles.

## Introduction

Understanding the intricate laws governing transport of nanomaterials, especially nanoparticles, through a porous medium has major implications in many different fields such as filtration technology, separation and water sanitation process^1,2^, geophysics^3,4^, biophysics^5^, and medicine^6^. For example, a major challenge encountered in nanomedicine development is improving NPs transport within interstitial tissues^6^. Interstitial tissues typically contain gel-like networks of entangled polymer chains with mesh sizes in the range of tens to hundreds of nanometers^7^, posing a critical constraint on the diffusion of nanomedicine. Large NPs (diameter > 100 nm) get trapped and cannot diffuse deep in the tissue, limiting their therapeutic efficacy. A straightforward solution is to use sufficiently small NPs, so that the surrounding network does not have any effect on them, and they diffuse without any constrictions in the background liquid media^8,9^. However, in several cases, it has been shown that NPs larger than the medium characteristic pore size can be far better drug carriers than smaller particles. For example, polymeric micelles with diameters ranging between 100–160 nm demonstrated long plasmatic circulation time and strong accumulation into tumors, whereas significantly less amount of smaller micelles penetrated inside tumoral tissues, even though these small micelles travelled much deeper in the tissues^10^. Strategies to facilitate NPs deep tissue penetration include NPs surface modification^11^, local transformation of the connective tissue or extracellular matrix^12^, and design of smart NPs responsive to local physicochemical stimulations^13,14^. Although these strategies have been effective in several cases, they are often designed for very specific situations, and cannot be integrated into one multipurpose drug delivery system^15^. Therefore, to date, improving NPs penetration through biological barriers is still an outstanding technological challenge.

Soft hydrogel NPs, i.e. NPs synthesized by crosslinking a hydrophilic polymer, possess attractive attributes as drug delivery systems. Recent in vivo studies showed increased circulation time^16^, lower immunogenicity^16^, and increased tissue penetration^17^ using hydrogel NPs compared to their hard counterparts. Longer circulation time is believed to result from two distinct mechanisms: a facilitated escape from the reticulo-endothelial system and a decreased uptake from the immune system, specifically from macrophages^16^. These observations suggest that soft NPs can diffuse through biological barriers and penetrate interstitial tissues more efficiently compared to hard NPs with their deformable nature^18–20^. Few mechanisms for the improved penetration of soft NPs have been proposed. Hendrickson et al. reported that hydrogel NPs can cross permeable membranes with pore size much smaller than the NPs diameter under high enough hydrostatic pressure^19^, a behavior that was ascribed to pressure-induced deformation of the NPs. Yu et al. reported that semi-elastic NPs can adopt an ellipsoidal shape when immersed in a hydrogel matrix, interacting strongly with the NPs and diffusing faster than hard spheres^20^.

To elucidate the diffusion mechanism of NPs in a porous medium, we compared the dynamics of soft and hard NPs in agarose hydrogels as a model system. We provide evidence that soft NPs diffuse much faster than hard spheres when dispersed in a hydrogel matrix. Such behaviour is reminiscent to their capacity to dynamically adjust their size under the influence of long-range interactions with their environment.

## Results

### Hard and soft NPs diffusivity in water

We used poly(N-isopropylacrylamide) (pNIPAM) hydrogel NPs as model soft hydrogel NPs. These particles have an elastic modulus ranging between 1–10 kPa and are known to be highly deformable under external stimuli such as pH, temperature, and osmotic pressure^21–23^. The diffusion coefficient of these NPs in agarose gels was compared to that of hard NPs (elastic moduli in the GPa range) made of polystyrene^24^ (PS) or gold^25^ (Au) of similar hydrodynamic radius.

Differential dynamic microscopy (DDM) was used to assess the dynamics of these particles in agarose solutions and gels. DDM allows high precision measurement of unlabelled NPs diffusion in a transparent media based on video microscopy^26–28^. This technique uses low-resolution movies to obtain the differential image correlation function *g*(*q,τ*), i.e. the power spectrum of the difference between images pairs separated by a delay time *τ*, at a spatial frequency *q* = 2*π*/*L*, with *L* being the length-scale of interest. Under appropriate imaging conditions, the experimentally measured function *g*(*q,τ*) is related to the intermediate scattering function (ISF) *f*(*q,τ*) ^[15]^ as

1 $$g\left( {q,\tau } \right) = A\left( q \right)[1 - f\left( {q,\tau } \right)] + B(q),$$

where *A*(*q*) and *B*(*q*) are the signal amplitude and instrumental noise, respectively. For diffusing spherical NPs, the ISF takes the generalized exponential form of $f\left( {q,\tau } \right) = e^{ - (\tau /\tau _R)^\beta }$, where *τ*_R_ is the relaxation time and *β* is the stretch exponent. For non-interacting monodisperse diffusing particles, *β* = 1, while interactions between particles and media and suspension polydispersity systematically lead to *β* < 1. From the relaxation time, *τ*_R_, the effective diffusion coefficient *D* = 1/*q*^2^τ_*R*_, and thus the hydrodynamic radius, *r*_*H*_, can be estimated using the Stokes-Einstein (SE) equation *D* = *k*_*B*_*T*/6*ηr*_*H*_, with *k*_B_ being the Boltzmann constant, *T* the absolute temperature and *η* the medium viscosity.

**Fig. 1:**
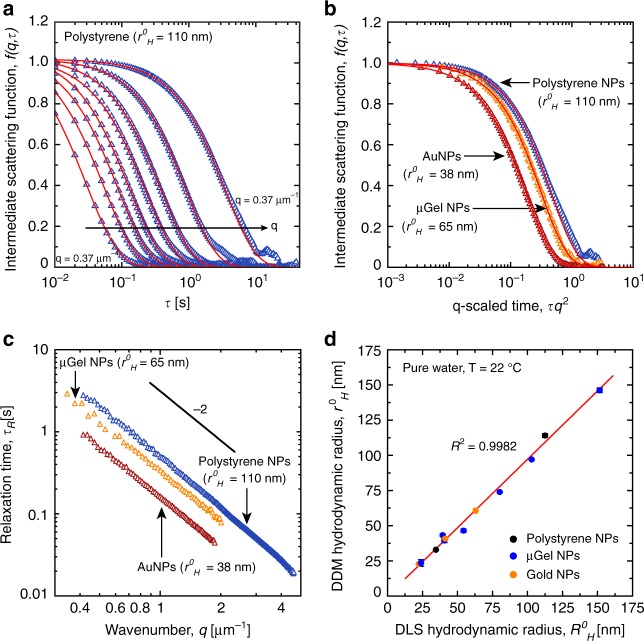
Dynamics of soft and hard nanoparticles in pure water at volume fractions *ϕ* < 0.1%. **a** DDM intermediate scattering functions extracted from *g(q,τ)* functions using Eq. (1), showing the dynamics over a large sample of *q*. **b** Intermediate scattering functions as a function of the spatial-frequency-scaled time for hard NPs (blue triangle, *q* = 0.37 μm^−1^, red triangle *q* = 0.57 μm^−1^) and soft NP (orange triangle, *q* = 0.63 μm^−1^). **c** Relaxation time *τ*_R_ vs *q* for different soft and hard NPs radii. Data shows the typical scaling relation between *τ*_R_ vs *q* with an exponent of −2. **d** Comparaison of the hydrodynamic radius obtained from DDM (*r*^0^_H_) and DLS (*R*^0^_H_). The red line is a linear least square fit to the data (slope = 0.972 ± 0.012) with its corresponding R^2^ value. Numerical values for hydrodynamic radius are provided in Supplementary Table 1

The dynamics of hard and soft NPs was first characterized in water as a reference medium. Figure 1a shows typical ISFs and their associated relaxation times (Fig. 1b) obtained by DDM in dilute hard and soft NP suspensions at volume fractions *ϕ* < 0.3%. Data reveal that *τ*_R_(*q)* follows a power-law decay with an exponent of −2, and the stretching factor is systematically superior to 0.9, as expected for pure diffusion of nanospheres. The NP hydrodynamic radius in diluted suspension, *r*^0^_H_, was quantified using the SE equation and cumulant analysis^27,29^. Figure 1c shows that *r*^0^_H_ values are quantitatively very close to values obtained by dynamic light scattering (DLS), *R*^0^_H_, which were obtained by the same cumulant analysis. A good agreement within 3% between *r*^0^_H_ and *R*^0^_H_ was obtained.

### Hard and soft NPs diffusivity in agarose solutions and gels

**Fig. 2:**
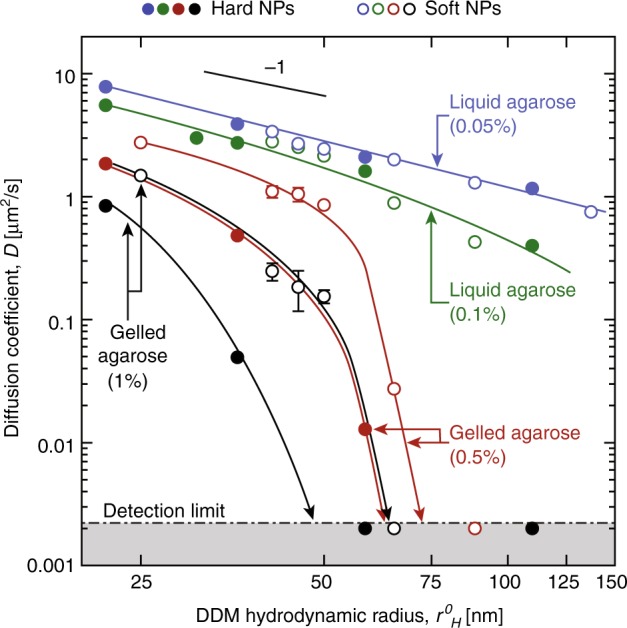
Diffusion of hard and soft NPs in agarose. Diffusion is compared at different concentrations of agarose (*C*_ag_ = 0.05, 0.1, 0.5, 1% w/w) versus the hydrodynamic radius *r*^0^_H_ measured in water by DDM. Solid lines are guides to the eye, while the grey area shows the region below the detection limit, also indicating NP immobilization. Each point is presented with an error bar that corresponds to an average of 5–7 DDM measurements on one sample and its standard deviation

Next, the effect of confinement on NPs dynamics in agarose solutions and gels of varying agarose concentrations, *C*_ag_, was investigated. The diffusion coefficient of soft NPs with *r*^0^_H_ = 25–130 nm was measured in parallel to hard NPs of similar radius *r*^0^_H_ = 22–110 nm at *ϕ* < 0.3% as shown in Fig. 2. The extraction process of *τ*_R_(*q)* from representative ISFs for all *C*_ag_ is shown in Supplementary Fig. 1 and described in Supplementary Notes 1. We confirmed for hard and soft NPs that *τ*_R_(*q*) follows the general scaling relationship *τ*_R_ *~ q*^−2^, over the range of agarose concentrations investigated, therefore allowing the calculation of an effective diffusion coefficient *D*_G_ of the NPs in the gels (see Supplementary Figs. 2 and 3). Alongside with *τ*_R_(*q*), a stretch factor, *β* ≈ 0.7, was measured in agarose gels, indicating that NPs have a quasi-diffusive motion hindered by the gel matrix, while *β* ≈ 1 in agarose solutions (Supplementary Fig. 4).

Below the gelation point of agarose (*C*_ag_ = 0.05–0.1%w/w), no difference between the diffusion coefficient of hard and soft NPs was observed (see Fig. 2). In this *C*_ag_ interval, the diffusion coefficients of hard and soft NPs measured by DDM quantitatively matched theoretical calculations using the standard form of the SE equation,$D_{\mathrm{G}} = k_BT/6\eta r_H^0$, suggesting that the decrease in the diffusion coefficient measured in liquid agarose with increasing *C*_ag_ can be solely attributed to the increase in viscosity of the medium. At *C*_ag_ = 0.1%w/w, which is close to the sol-gel transition of agarose (see Supplementary Fig. 5, Supplementary Methods 1 & Supplementary Notes 2 for corresponding rheology experiments), *D*_G_ of soft and hard NPs with *r*^0^_H_ > 75 nm significantly deviated from SE predictions (see Fig. 2, green symbols), highlighting the appearance of interactions between NPs and the polymer matrix. For spherical diffusers in dilute and semi-dilute polymer solutions, Phillies et al.^30,31^ have proposed a general expression for the reduced diffusion coefficient *D*_G_*/D*_0_ = exp(*-μ C*_ag_^*ν*^), where *D*_0_ is the diffusion coefficient of the NP in pure water and μ ~ *r*_H_^*δ*^ *M*_w_^*γ*^. Here, *M*_w_ is the agarose molecular weight, *γ*, *ν*, and *δ* are scaling factors (*M*_w_^*γ*^ is constant in our experiments). Analysis of the data using Phillies equation provides a value of *δ* = 0.8 ± 0.16 for both hard and soft spheres. If agarose chains were freely moving in solution, a value of *δ* = 0 would have been expected^30,31^. Therefore, in this regime agarose chains are strongly interacting with NPs and alter their dynamics. More importantly, the fact that similar scaling parameter, *ν* ≈ 0.75, was found for hard and soft NPs indicates that their dynamics can be fully predicted knowing *r*_H_, which determines the value of *μ*.

In the gel regime (*C*_ag_ ≥ 0.5% w/w), the diffusion of the soft and hard NPs was slowed down by the polymer matrix due to gel-NPs interactions, but soft NPs were found to exhibit significantly faster diffusion compared to hard NPs (see Fig. 2, red and black symbols). Comparing the diffusion coefficient *D*_G_ of hard and soft NPs of similar size (*r*^0^_H_ = 62 and 50 nm, respectively), *D*_G_ of soft NPs was found to be nearly two orders of magnitude higher than hard NPs (*D*_G_ = 0.853 µm^2^/s for soft NPs compared to *D*_G_ = 0.013 µm^2^/s for hard NPs in *C*_ag_ = 0.5% w/w agarose, see Fig. 2). Such a large difference in dynamics was ascribed to a change in particle size under the influence of long-range electrostatic interactions.

### Osmotic pressure in agarose gels

To confirm this hypothesis, osmotic deswelling of the soft NPs, originating from the presence of free (un-crosslinked or dangling) agarose chains, was first ruled out^32–34^. The osmotic pressure, *Π*_osm_, inside *C*_ag_ = 1% w/w agarose gels was estimated by the dialysis bag method^35–37^ to be 89 Pa (see Supplementary Methods 2 and Supplementary Notes 3 for details), which is of the same order as *Π*_osm_ *≈* 0.4 kPa reported for *C*_ag_ = 1% w/w molten agarose solutions^32–34^ at *T* = 37 °C. This value is about two orders of magnitude smaller than the osmotic pressure needed to induce a 30% change in NPs radius in presence of Ficoll^TM^ 400 solutions (*Π*_osm_ = 9.1 kPa, see Supplementary Fig. 6)^23,38,39^, eliminating the potential contribution of osmotic stress from free agarose molecules to the change in particle size originating.

### Hydrodynamic and electrostatic contributions to diffusivity

To quantify the possible contributions of electrostatic and hydrodynamic interactions to the dynamics of the NPs in the gels, the theoretical framework developed by Kang et al.^9^ was used to analyze the data presented in Fig. 2. In this framework, hydrodynamic and electrostatic interactions are considered to be the sole interactions controlling the diffusion of spherical NPs in a percolating network of randomly oriented cylinders. The reduced diffusivity *D*_*G*_*/D*_*0*_ is expressed as the product of two separate terms, one for each interaction, as shown in Eq. (4)

4 $$\frac{{D_G}}{{D_0}} = \frac{1}{{1 + \alpha _{iso}^h{\mathrm{\varphi }}_{\mathrm{f}}}}\frac{1}{{1 + \alpha _{iso}^s{\mathrm{\varphi }}_{\mathrm{f}}}}.$$

The hydrodynamic interaction term is represented by the parameter $\alpha _{iso}^h$, while electrostatic interactions are contained in $\alpha _{iso}^s$. The parameter *φ*_f_ represents the agarose volume fraction, which varies between 0.0049 and 0.0098 in Fig. 2. The coefficient $\alpha _{iso}^h$ for hydrodynamic interactions is expressed as a function of the hydrodynamic screening length *κ*^−1^ and the dimensions of both the particles and the cylinders in Eq. (5),

5 $$\alpha _{iso}^h = - \frac{{\kappa r_H^0}}{{\left( {\kappa d_f} \right)^2ln\left\{ {\kappa d_f} \right\}}}\left[ {\frac{{64}}{{10}} - \frac{{85}}{{10000}}\kappa L - \frac{{33}}{{10000}}\left( {\kappa L} \right)^2} \right],$$

where *L* is the length and *d*_f_ is the diameter of the cylinders (the agarose fibres in the present case, *L* = 500 nm^40^, *d*_f_ = 3.8 nm^41,42^). The coefficient $\alpha _{iso}^s$ for electrostatic interactions is expressed in terms of the Debye-Hückel screening length *κ*_Q_^−1^ in Eq. (6),

6 $$\begin{array}{l}\alpha _{iso}^s = \frac{2}{3}\left( {1 + \frac{{2r_H^0}}{{d_f}}} \right)^2\hfill\\ {\kern 2pc}\times\left[ \begin{array}{l}1 + 2\frac{{l_B}}{L}\frac{{Z_cZ_f}}{{\left[ {1 + \kappa _Qr_H^0} \right]\left[ {1 + \frac{{\kappa _Qd_f}}{2}} \right]}} \frac{{22}}{{\left( {\kappa _Q\left[ {r_H^0 + \frac{{d_f}}{2}} \right]} \right)^2 + 25\kappa _Q\left[ {r_H^0 + \frac{{d_f}}{2}} \right] + 10}}\end{array} \right],\end{array}$$

where *l*_B_ is the Bjerrum length, *Z*_C_ and *Z*_f_ are the surface charge of the colloid and the agarose fibres, respectively. The surface charge *Z*_C_ of the colloids was estimated from zeta potential measurements (see Supplementary Table 1 & Supplementary Notes 4 for details) using Makino et al. equation^43^. The surface charge of the agarose fibres *Z*_f_ was determined using Buffle et al. and Johnson et al. considerations^42,44^, which gave a value of *Z*_f_ = −40. The hydrodynamic screening length, *κ* ^−1^ was calculated based on $\kappa ^{ - 1} = {\mathrm{A}}\varphi _f^{ - \mu }$, with the hydrodynamic constant *Α* = 0.33 and the de Gennes polymer constant *μ* = 0.75 for soft polymer chains^45,46^.

**Fig. 3:**
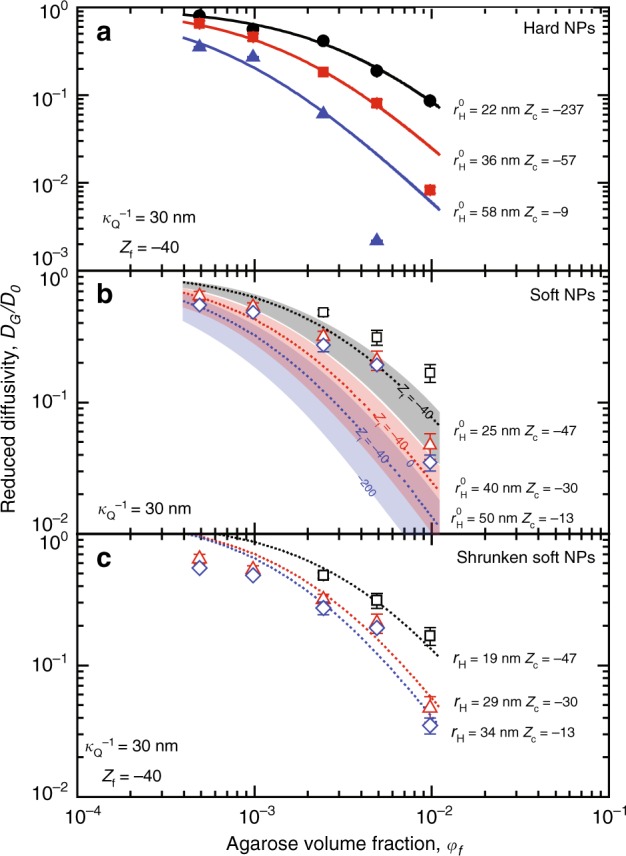
Experimental reduced diffusivities and theoretical prediction using Kang *et al*. model. **a** Data for hard NPs; **b** Reduced diffusivity of soft NPs. The theoretical curves were produced considering no change in particle size (*r*_H_ = *r*^0^_H_). Also presented are different simulated curves, using Eq. (4), fallen into the highlighted areas calculated for different values of *Z*_f_ to demonstrate that electrostatic interactions alone cannot account for the increased diffusivity of soft NPs. **c** Reduced diffusivity of soft NPs (dotted lines) calculated assuming *r*_H_ = *αr*^0^_H_. Data points and their error bars correspond to the average of 5–7 DDM measurements on one sample and their standard deviation

Figure 3a shows the evolution of the reduced diffusivity of hard NPs (*ϕ* < 0.01%) as a function of the agarose volume fraction in the gel, *φ*_f_. The reported theoretical curves (Eq. (4)), which were obtained with no fitting parameter, accurately describe the experimental data, confirming the validity of the estimated values used for the gel pore structure and for colloids and agarose fibres surface charges. Using the same set of parameters for the agarose gel and the corresponding parameters for the soft NPs did not lead to a similar agreement between theory and experiments (Fig. 3b). The measured diffusion coefficients were systematically higher than the predicted values, if the soft NPs radius was kept constant and equal to *r*^0^_H_. It is interesting to note that the electrostatic interaction contribution by itself cannot account for the observed discrepancy. Indeed, increasing (or decreasing) the values of *Z*_f_ or *Z*_C_ significantly decreases the predicted reduced diffusivity and does not provide any conciliation with experimental data (see Fig. 3b). On the other hand, using *r*_H_ as the sole free parameter in Eq. (4) allows obtaining excellent agreement between theoretical and experimental values at high *C*_ag_ (see Fig. 3c). At low *C*_ag_, the particles are highly swollen and have a different *r*_H_ compared to the NPs at high *C*_ag_, which is not accounted for in the Kang *et al*. model. The *r*_H_ values obtained for all the tested soft NPs were significantly smaller than *r*^0^_H_ at high agarose volume fractions, indicating shrinkage of the NPs due to confinement. At *φ*_f_ = 4.98 × 10^−3^, the shrinking ratio $\alpha = r_H/r_H^0$ was within 0.59 to 0.77, depending on the soft NPs size, which is consistent with *α* = 0.67 ± 0.12 obtained when exposing the soft NPs to 50 mg/mL Ficoll^®^ solutions. Soft hydrogel NPs have been reported to exhibit a stiff core and a fuzzy corona (~1/3 of the NP radius), resulting from the radial decay of crosslinker concentration from their center^39,47^. Therefore, it is reasonable to conclude that the measured value of *α* ≈ 2/3 in agarose gels and Ficoll^®^ solutions is the result of compressing the fuzzy corona.

The discussed experiments above were performed in pure water after extensive dialysis to remove ions (see materials and methods). The measured conductivity in the gel indicated an equivalent monovalent ion concentration of 10^−4^ M, which corresponds to a Debye length *κ*_Q_^−1^ ≈ 30 nm (see Supplementary Table 2), indicating that long-range repulsive electrostatic interactions are present between the particles and the hydrogel matrix. Tuning the value of *κ*_Q_^−1^ using monovalent ions had a strong impact on the dynamics of the soft NPs and hard NPs. By increasing the salt concentration ([NaCl] = 10^−1^ M, *κ*_Q_^−1^ ≈ 1 nm), the diffusion coefficient of the soft NPs was found to exactly follow the theoretical values predicted by Eq. (4) using *r*^0^_H_ (and not *r*_H_, see Supplementary Fig. 7), after accounting for the changes in gel structural parameters. Eq. (4) predicts an increase in the reduced diffusivity *D*_*G*_*/D*_*0*_ of ~ 60–200% (depending on the particle size and charge) when increasing salt concentration from 10^−4^ to 10^−1^ M, assuming no change in the particle size. However experimentally *D*_G_*/D*_0_ increased only by 10% for the soft NPs, which can only indicate that the NP size has dramatically increased in the gel (see Supplementary Notes 5). Note that prior to these tests, it was verified by time-dependent measurements of *r*_*H*_ in saline solutions (no agarose) that no aggregation of the soft NPs occurs up to 1 M NaCl in water. Also, adhesive interactions between the hydrogel NPs and agarose can be ruled out since no change in *r*_H_ (*r*_H_ = *r*^0^_H_) was measured in liquid agarose solutions, indicating that agarose chains do not adsorb on the NPs. Therefore, the presented results confirmed that in high salinity solutions, the soft NPs embedded in agarose gel fully recovered their original size (*r*_H_ = *r*^0^_H_).

### Electrostatic interactions in the EDL

Therefore, it appears that the observed change in the diffusion coefficient, thereby in the particle size, is mediated by long-range electrostatic interactions between the NPs and the agarose fibres, since interactions between particles is non-existent in suspensions at such low *ϕ* (see Supplementary Methods 3 and Supplementary Fig. 8). Agarose gel fibres are negatively charged (*Z*_f_ = −40 charge per fibre), which creates an ion cloud surrounding fibres similar to the ion cloud surrounding the NPs to form the so-called electrostatic double layer (EDL)^48,49^.

**Fig. 4:**
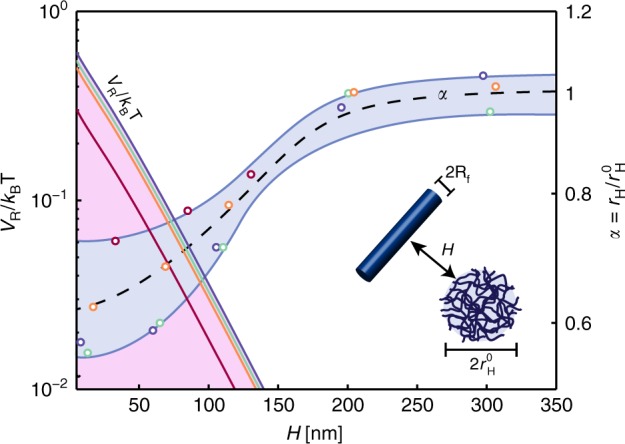
Electrostatic interactions in the EDL.Theoretical reduced interaction potential, *V*_*R*_*/k*_*B*_*T*, between a sphere and a fibre (left axis) and experientally measured NPs shrinking ratio, *α*, (right axis) as a function of the surface-to-surface distance *H*. Experimental values for *α* were collected at different agarose concentrations *C*_ag_, which were used to calculate *H* (see Supplementary Notes 6). The different colors used for the symbols (circles refer to *α*) and curves (which refer to *V*_*R*_*/k*_B_*T*) correspond to different values of *r*^0^_H_ (red: *r*^0^_H_ = 25 nm, orange *r*^0^_H_ = 40 nm, blue *r*^0^_H_ = 45 nm, green *r*^0^_H_ = 50 nm)

In support of this hypothesis, the overlapping of soft NPs and agarose fibres EDLs was investigated by calculating the electrostatic interaction potential between a single agarose fibre and a soft NP (Fig. 4). The electrostatic interaction potential *V*_R_ between a spherical NP and an infinite cylinder in water was calculated using^50^

7 $$\begin{array}{l}V_R = - 4{\it{\epsilon }}\sqrt {2\pi \kappa R_f} {\int}_0^{r_H^0} {x\sqrt {\frac{x}{{\left( {r_H^{02} - x^2} \right)\left( {R_f + x} \right)}}} } \hfill\\ {\kern 1.8pc}\times\left\{ {\begin{array}{*{20}{c}} {\left( {\frac{{\psi _{0C} + \psi _{0f}}}{2}} \right)^2Li_{1/2}\left[ { - e^{ - \kappa _Q\left( {H + r_H^0 - x} \right)}} \right]} \\ { + \left( {\frac{{\psi _{0C} - \psi _{0f}}}{2}} \right)^2Li_{1/2}\left[ { - e^{ - \kappa _Q\left( {H + r_H^0 - x} \right)}} \right]} \end{array}} \right\}dx,\end{array}$$

where *R*_f_ is the cylinder radius (agarose fibre), *ε* is the water permittivity, *L*_i_ is the polylogarithm function, *H* is the particle to fibre surface-to-surface distance and *ψ*_0_ is the surface potential of the NPs (C) or the fibres (F) whose expression for the NPs is given by

8 $$\psi _0 = \frac{{4k_BT}}{e}{\mathrm{tanh}}\frac{{e\psi _d}}{{4k_BT}},$$

where *ψ*_d_ is the zeta potential and *k*_B_*T* is the thermal energy. For the fibre, the surface charge density, *σ*, is related to the surface potential through^51^:

9 $$\sigma = \frac{{2\it{\epsilon}\kappa _Qk_BT}}{e}\sinh \left( {\frac{{e\psi _0}}{{2k_BT}}} \right)\left[ {1 + \left( {\frac{1}{{\beta ^2}} - 1} \right)\frac{1}{{{\mathrm{cosh}^{2}}\left( {\frac{{e\psi _0}}{4k_BT}} \right)}}} \right]^{1/2}.$$

Here,

10 $$\beta = \frac{{K_0(\kappa _Qd_f/2)}}{{K_1(\kappa _Qd_f/2)}},$$

where *K*_n_(*z*) is the modified Bessel function of the second kind of order n.

Plugging the experimental parameters in Eq. (7), theoretical interaction potential curves were generated for NPs of different sizes in agarose gels of different *C*_ag_ (which defines the pore size in Supplementary Table 3 and the separation distance *H* as detailed in Supplementary Notes 6) and compared to the shrinking ratios *r*_H_/*r*^0^_H_ measured by DDM. As shown in Fig. 4, the shrinking ratio, *α,* of the soft NPs decreases from 1 to 0.6 when the particle-cylinder distance reaches the critical distance at which the repulsive electrostatic interaction begins (onset of interaction). This transition occurs for *H* ranging between 100 and 150 nm depending on the value of *r*^0^_H_. The close correspondence between the onset of shrinking and the onset of electrostatic interaction is consistent with the hypothesis that EDL overlapping plays a major role in the shrinking of the NPs. The predicted value of the repulsive potential may appear rather small to create the required osmotic pressure for NPs shrinking in the gel, but it should be noted that the present calculation is accounting for the interaction between only one fibre and the NP. It is expected that many other fibres can simultaneously interact with the NP to create a much stronger pressure capable of triggering particle shrinking. The energy, *U*, required to compress an elastic nanosphere of elastic modulus *E* = 1−10 kPa to 66% of its original size is *U* = 1–10 *k*_B_*T* (see Supplementary Notes 7 for the calculations), which would approximately correspond to the interaction energy between 10–100 fibres and the NP.

## Discussion

We have shown that hydrogel NPs shrinking occurs under confinement in gels, and found that such behaviour is due to the interactions between overlapping electrical double layers. Thanks to their deformable nature, hydrogels NPs shrink and diffuse 1 or two orders of magnitude faster than hard NPs under weak interaction pressure. Such peculiar ability may be triggered not only by electrostatic interactions, which in the present study were tuned by saline concentration, but in principle by any long-range interaction force. Different examples of such long-range forces exist in the biological realm, especially those involving thermal fluctuations such as undulation forces originating from cell membrane undulation or steric polymer forces from for example pericellular glycosylated moieties^52,53^. For example, compressive forces originating from membrane undulations^52^ are proportional to *(k*_B_*T)*²*/k*_b_*H*³, (where *k*_b_ = 0.1–10 *k*_B_*T* is the membrane bending modulus, and *H* is the separation distance) are of the order of 10^−1^ – 10^3^ Pa for *H* in the range of 100–20 nm, which is sufficient to trigger soft NP compression. In the same line, considering a typical contour length of glycosylated moieties of 50–700 nm^54^ and in some cases up to 11 μm^55^, the Alexander – de Gennes steric interaction pressure generated by these moieties on a particle is written as *P*(*H*) = 100*Γ*^3/2^*k*_B_*T*exp*(-*π*H/t)*, with *Γ* being the number of chains per unit area, and *t* the effective thickness of an adsorbed layer of moieties. Considering average values of 400 nm and 0.001 nm^−2^ for t and *Γ*^56^, the resulting steric pressure is of the order of 10^2^ – 10^5^ Pa, again sufficient to produce soft NP shrinking.

By taking advantage of their distinct ability to diffuse faster in complex crowded media, soft NPs can be invaluable assets when used as drug carriers for example. The results presented in this study echo the ever-increasing in vivo evidences showing that soft deformable NPs^16,57^ have the unusual capacity to penetrate more deeply into soft porous tissues. The soft NP shrinkage in this study was attributed to the pressure from the long-range electrostatic interactions. Although electrostatic interactions are short-range in vivo, other long-range interactions in biological environment could generate similar pressures. Therefore, careful attention should be paid to the design of the NPs mechanical properties and deformability in order to control their biological fate.

## Methods

### Chemicals

N-Isopropylacrylamide (NIPAM), N,N’-methylene-bis(acrylamide) (BisA), methacrylic acid (MAA) with 250 ppm inhibitor MEHQ and sodium dodecyl sulfate (SDS) ~95% were purchased from Sigma–Aldrich (Oakville, ON, Canada). Ficoll^TM^ 400 (molecular weight ≈ 400 kDa) and agarose (low melting point) were purchased from Fisher Scientific (Montréal, QC, Canada).

### Hard Nanoparticles preparation

Gold NPs of hydrodynamic radius *R*_H_ = 45, 75, and 125 nm (core diameter of 20, 50 and 100 nm) were purchased from Nanocomposix (San Diego, CA, USA). PS NPs (*R*_H_ = 60 and 220 nm) were purchased from Nanospheres. Silica NPs (Ludox TM-40) were purchased from Sigma–Aldrich (Oakville, ON, Canada). The nanoparticles were diluted with Milli-Q^®^ water as needed.

### Microgel synthesis

Microgel synthesis was performed as previously reported^58,59^. Typically, the reaction was held in a three-neck flask under argon flow and mechanical stirring (~300 rpm). NIPAM was dissolved with MAA (NIPAM:MAA ratio of 100:0 and 95:5), BisA (5.3 mol% total monomer), and SDS in degassed water. Microgels smaller than *r*^0^_H_ = 92.5 nm were synthesized without MAA and the largest microgel (*r*^0^_H_ = 140 nm) with 5% molar MAA.

SDS concentration 0.87, 1.73, 3.47, 4.33, 8.67 mmol/L was used to tune microgel sizes (smaller microgels were produced by increasing SDS concentration). The reaction was initiated at 65 °C by adding APS (2.7 mmol/L) with a subsequent rise in the temperature to 75 °C for 4h30. Upon completion, the NPs suspension was removed from the flask and allowed to cool at room temperature. Each batch was dialyzed three times using Spectra/Por Tube-A-Lyzer Dynamic Dialysis Device (100 kDa MWCO) against Milli-Q water (∼60–70 mL of particle suspension for 20 L of water for each dialysis cycle). NPs concentration within each batch was determined by freeze-drying 1.5 mL of the microgel suspension and weighting its dry mass. Colloidal microgels were stored at 4 °C until further use.

### Dynamic light scattering

DLS data presented in Fig. 1 were obtained with a Malvern Zetasizer NanoZS (Malvern Instruments, UK). Experiments were performed at a single scattering angle *θ* = 173^o^ at a temperature of 22 °C for dilute suspensions (*ϕ* < 0.5%, details of the calculation in Supplementary Methods 4). Using Malvern software (cumulant analysis), the NP hydrodynamic radius (*R*^0^_H_) was obtained by identifying the peak of the intensity-weighted particle radius distribution.

### Differential dynamic microscopy (DDM)

An upright microscope (Olympus BX81, Japan) equipped with a high acquisition speed camera (Hamamatsu Orca-Flash 4.0 V3, Japan) was used for the acquisition of videos of NPs suspensions. Videos were recorded using phase-contrast imaging using ×20 or ×40 magnification (Olympus Plan, NA = 0.4 (Ph1) and NA = 0.65 (Ph2), respectively), a framerate ranging from 20 to 300 frames per seconds, and an image binning of 1 × 1 or 2 × 2 within a region of interest of 512 × 512 pixels. These parameters were adjusted to optimize the signal amplitude and to access particle dynamics over an adequate *q* range and time-scale for each NP. Images were recorded in glass capillaries (Vitrocom, Canada) of thickness 0.4 mm filled with nanoparticle suspension (~180 µL) and sealed using petroleum jelly. Videos were recorded at least five times at three different positions for each capillary.

In some instances, the presence of a second decay in the autocorrelation function was observed at longer time, presumably due to larger aggregates or very slow relaxation of the gel. For these specific measurements, we used a double generalized exponential model to extract the short-time process:

11 $$\begin{array}{l}g\left( {q,\tau } \right) = A1\left( q \right)\left( {1 - e^{ - \left( {\frac{\tau }{{\tau _{R1}}}} \right)^{\beta 1}}} \right) + A2\left( q \right)\left( {1 - e^{ - \left( {\frac{\tau }{{\tau _{R2}}}} \right)^{\beta 2}}} \right) + B\left( q \right),\end{array}$$

with indices 1 and 2 corresponding to the short and long-time processes, respectively.

### NPs diffusion measurements in agarose

To prepare NP suspensions in agarose, stock solutions of agarose were prepared at a concentration of 40 mg mL^−1^. Agarose was dissolved in hot water using a common microwave oven. The stock solution was diluted accordingly to prepare agarose solutions of 0.05, 0.1, 0.5, and 1% (w/v) using warm diluted suspensions of NPs. The resulting suspensions was injected in a rectangular capillary as described and was let to cool-down at room temperature for at least 24 h. Preliminary data were obtained during the cooling time to ensure that the gel was stabilized, and that the particles were not aggregating.

## Supplementary information

Supplementary Information

## Data Availability

The data that support the findings of this study are available from the corresponding author upon request.
