# Supplementary material for: Spontaneous shrinking of soft nanoparticles boosts their diffusion in confined media
Source: Nat Commun. 2019 Sep 20;10:4294. doi: 10.1038/s41467-019-12246-x (PMC6754464; doi:10.1038/s41467-019-12246-x)
Supplement: Supplementary file 1 — Supplementary Information [file 41467_2019_12246_MOESM1_ESM.pdf]

Supplementary Information for "*Spontaneous Shrinking of Soft Nanoparticles Boosts their Diffusion in Confined Media*"

Supplementary Figures

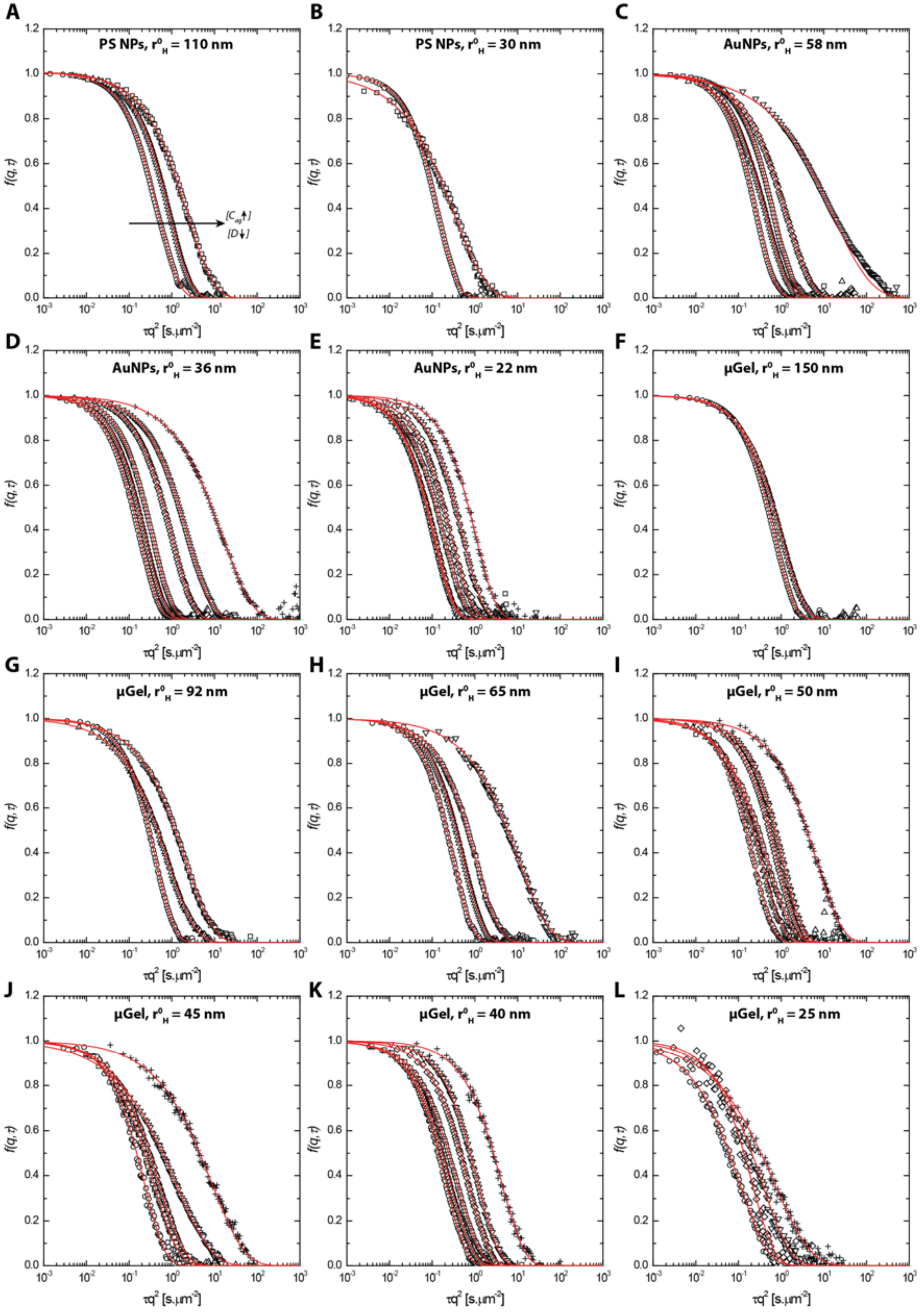

Supplementary Figure 1. Intermediate scattering function, $f(q,\tau)$, extracted from DDM autocorrelation functions, $g(q,\tau)$, plotted as a function of q^2-scaled time ($q^2\tau$) for different particles (A-L) and agarose concentrations present in the main text figure 1, 2 and 3. Each agarose concentration, notably, 0 w/v% (circles), 0.05 w/v% (upward triangles), 0.1 w/v % (squares), 0.25 w/v% (diamonds), 0.5 w/v% (downward triangles) and 1 w/v% (crosses) are presented and fitted according to the generalized exponential $f(q,\tau) = e^{-(\tau/\tau_R)^\beta}$. Some ISFs had a second relaxation mode and the ISFs were corrected using Eq. 11 for which the second relaxation process was occurring between $\tau_{R2} \approx 10$ - 200 s with $\beta_2 \approx 0.6$-2.5. This very slow process was found consistent to a small drift of the sample observed in those few videos. Samples that were affected by the thermal drift were: agarose 0.05% (AuNPs 38 nm, AuNPs 22 nm, μGel 150 nm, μGel 50 nm, μGel 45 nm), agarose 0.1% (μGel 50 nm), agarose 0.25% (AuNPs 58 nm, μGel40) agarose 0.5% (μGel 50 nm, μGel 45 nm, μGel 25 nm), agarose 1% (μGel 25 nm). The presented ISFs here are only representative examples of approximately 60 000 to 100 000 generated ISF in DDM in this study.

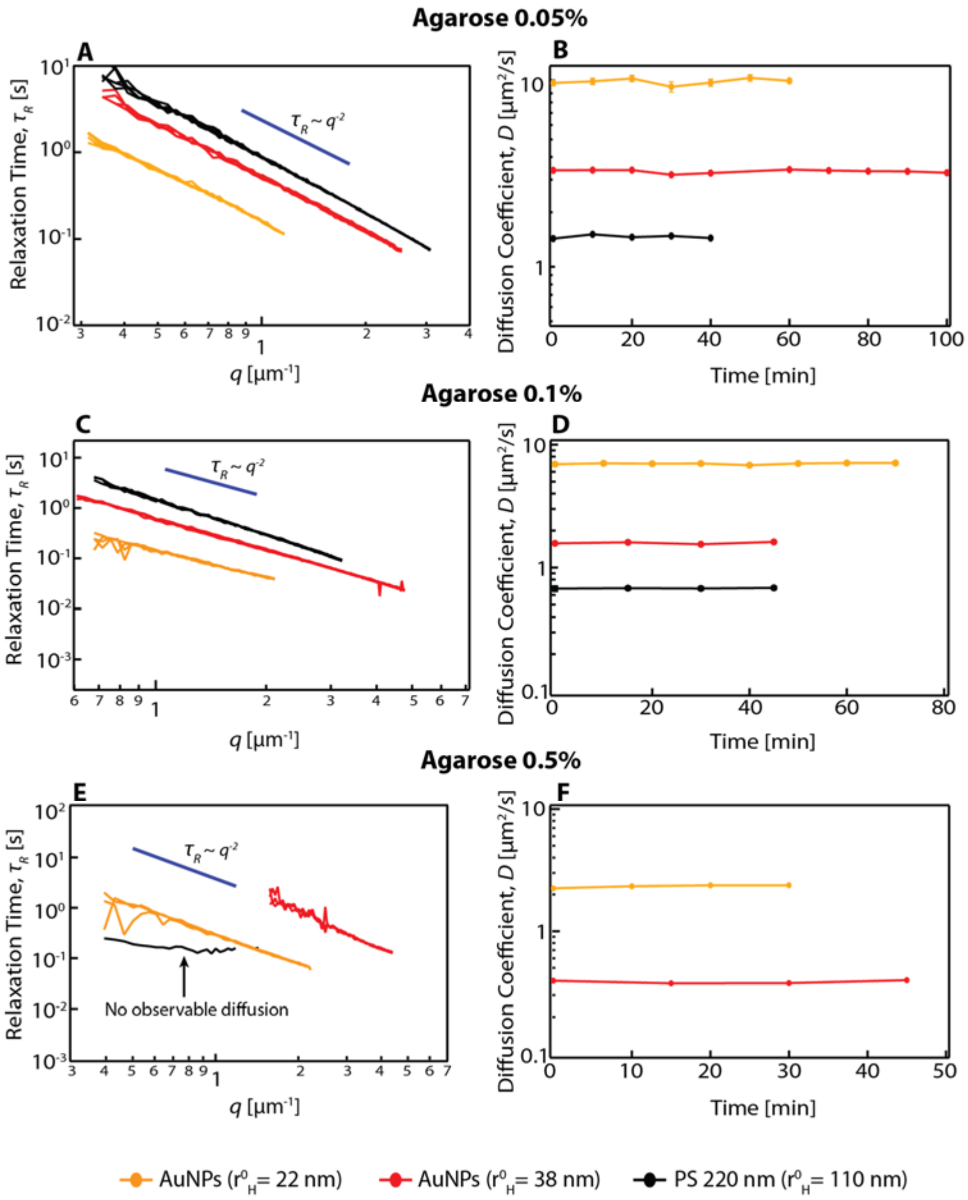

Supplementary Figure 2. DDM measurements of τ_R for hard NPs in agarose gel. (A, C, E and G) Relaxation time expressed as a function of q in agarose. The dynamics of NPs in solution is compared to the expected diffusion behavior $D = \tau_R(q)^{-1}q^{-2}$ (C). (B, D, F) Measurement of the diffusion coefficient of hard NPs 16h after gelation of the agarose gel.

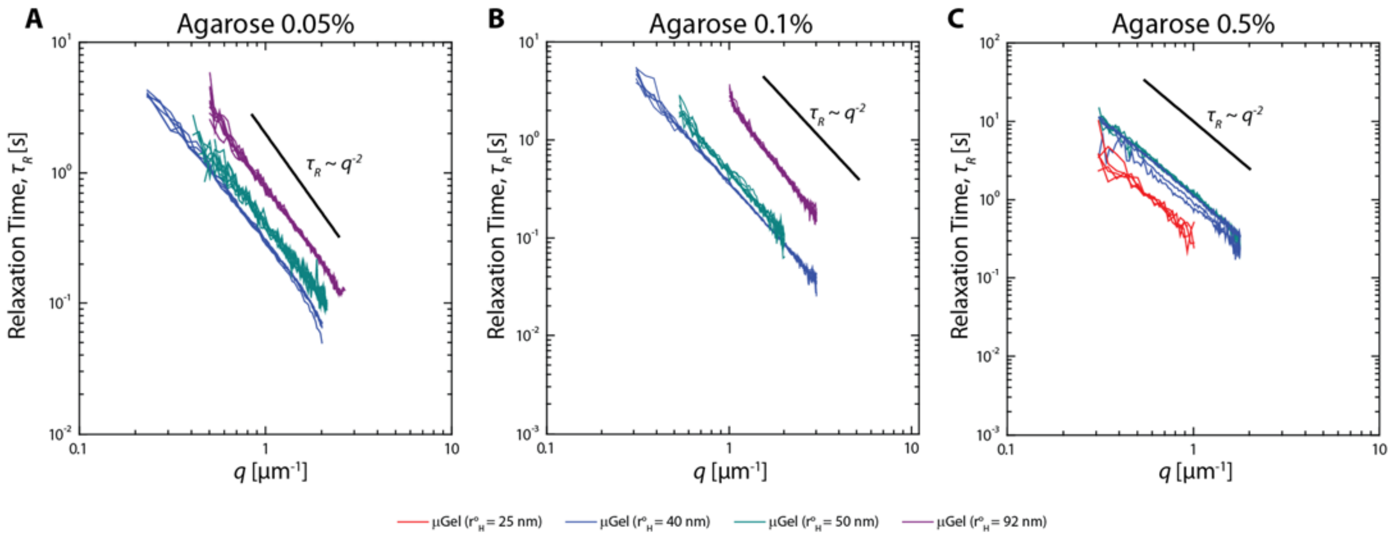

Supplementary Figure 3. DDM measurements of τ_R for soft NPs in agarose gels: A) C_{ag} = 0.05%, B) C_{ag} = 0.1% and C) C_{ag} = 0.5% w/w. Large soft NPs (r^0_H = 92 nm) were not diffusing in agarose at C_{ag} > 0.5%, and were not shown in panel C but were replaced by small soft NPs (r^0_H = 25 nm). Lines in red, blue, teal and purple are NPs with r^0_H = 25, 40, 50 and 92 nm respectively.

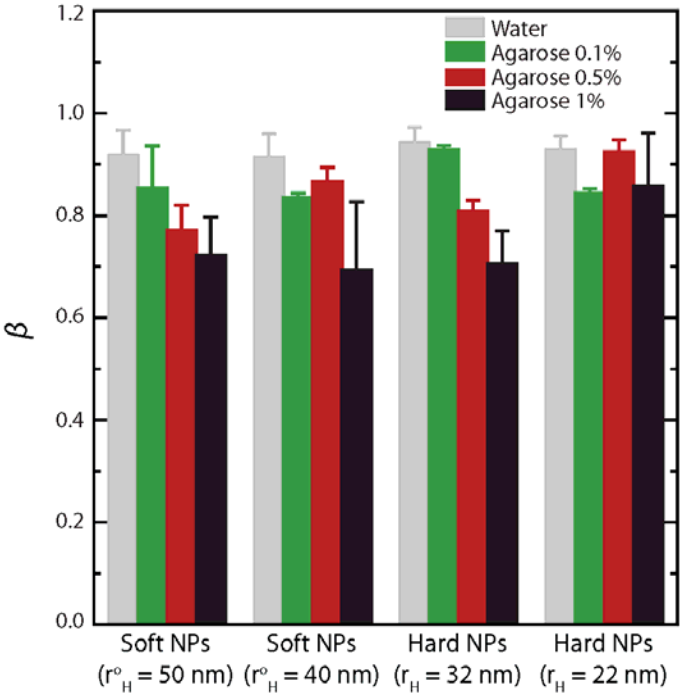

Supplementary Figure 4. Stretch exponent of the ISF for hard and soft NPs in water (gray), agarose C_{ag} = 0.1% (green), 0.5 % (red) and 1 % (black).

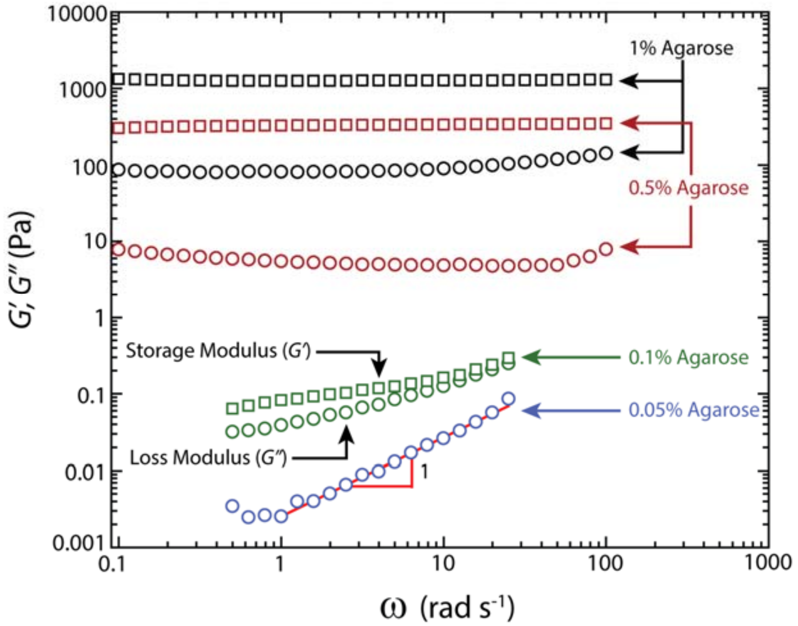

Supplementary Figure 5. Representation of the rheological properties of the agarose solutions and gels in pure water at ambient temperature (T≈22ºC). Squares represent the storage modulus (G') and circles represent the loss modulus (G'') for each agarose concentrations. Storage modulus data for 0.05% agarose was not shown in this graph because the values were below instruments detection limit.

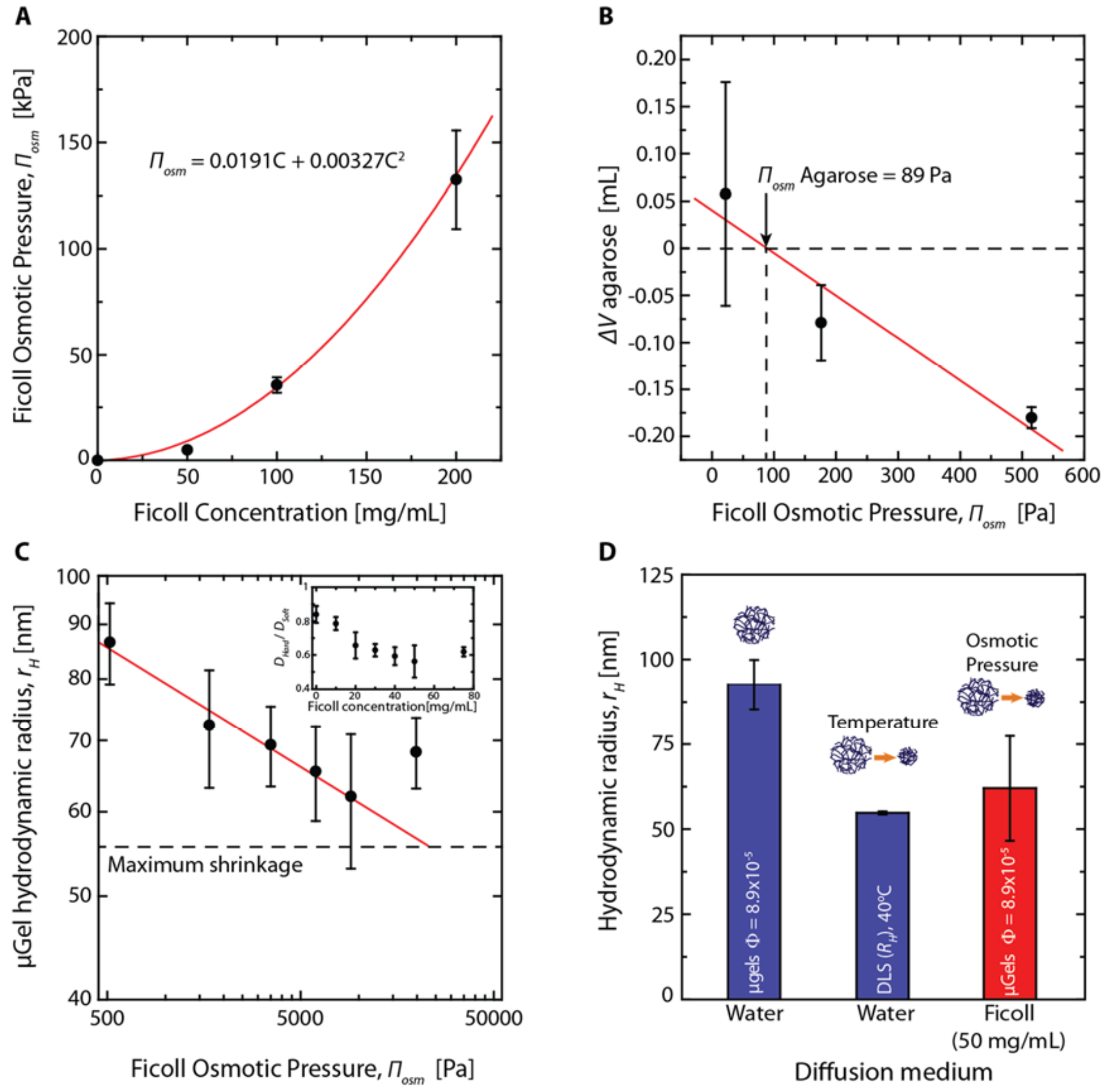

Supplementary Figure 6. (A) Osmotic pressure of Ficoll solutions of different concentrations. (B) Volume change of agarose gel (C_{ag} = 1% w/w) at equilibrium (or 7 days) at different Ficoll osmotic pressure. Red line is a linear fit: $\Delta V = 0.0403 - 0.00045\ \Pi_{osm}$ (C) Shrinkage of microgels with concentration of Ficoll osmotic pressure and in inset, the ratio of the coefficient of diffusion of hard and soft NPs for the different Ficoll concentrations. The red line corresponds to the power law fit: $r_H = 342\ \Pi_{osm}^{-1/9}$ (D) Comparison of different stimuli triggering shrinking of the microgels.

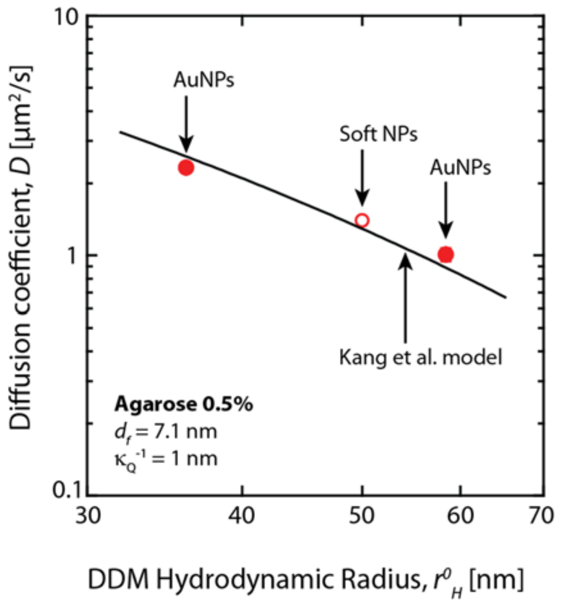

Supplementary Figure 7. Diffusion coefficient of hard and soft nanoparticles in agarose 0.5 % w/w in presence of 10^{-1} M NaCl. The line is the theoretical prediction of Kang et al. (Eq. 4) using r^0_H as the particle radius.

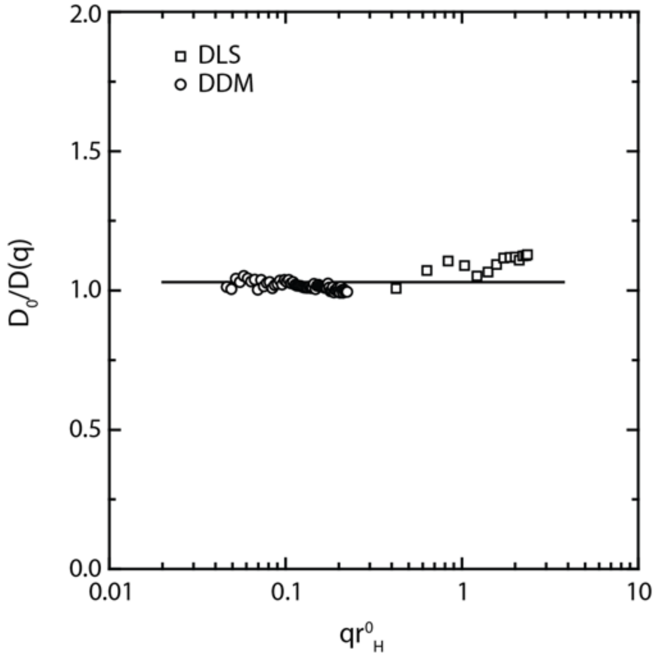

Supplementary Figure 8. Dependence of the dynamics $D_0/D(q)$ for r^0_H = 92 nm microgel at ϕ = 0.03% measured by DDM (circles from 0.04 – 0.25 qr_H^0) and DLS (squares from 0.4 – 3 qr_H^0). Here, q is normalized by the dilute radius r^0_H of the particle and the peak structure factor would be expected to be located at $qr^0_H \approx 1$ which does not appear in this graph.

Supplementary Tables

Supplementary Table 1. Hydrodynamic radius by DDM and DLS and Zeta potential of hard and soft NPs measured by DLS at a 4 mM NaCl concentration

**Type of NP**	**DDM, r^0_H (nm)***	**DLS, R^0_H (nm)***	**DDM, PdI**	**DLS, PdI**	**ZP at 22ºC** (mV)	**ZP at 38ºC** (mV)
**µGel r^o_H = 140 nm**	146 ± 2	152 ± 1	0.01	0.07	N/A	N/A
**µGel r^o_H = 92 nm**	97.0 ± 0.3	103 ± 1	0.03	0.02	-1.6 ± 0.3	-11.8 ± 1.8
**µGel r^o_H = 65 nm**	74.0 ± 0.5	80.2 ± 0.6	0.04	0.08	-2.7 ± 1.1	-9.0 ± 2.9
**µGel r^o_H = 50 nm**	46.5 ± 1.1	54.3 ± 0.2	0.16	0.14	-1.3 ± 0.2	-7.2 ± 2.9
**µGel r^o_H = 45 nm**	43.3 ± 0.9	39.5 ± 0.3	0.18	0.25	-1.4 ± 0.2	-8.4 ± 1.6
**µGel r^o_H = 40 nm**	40.8 ± 0.7	41.2 ± 0.2	0.13	0.22	-3.1 ± 0.6	-7.6 ± 1.7
**µGel r^o_H = 25 nm**	23.5 ± 2.4	23.9 ± 0.6	0.26	0.25	N/A	N/A
**AuNPs r^o_H = 22 nm**	22.8 ± 0.1	22.3 ± 0.1	0.12	0.11	-6.5 ± 1.2	N/A
**AuNPs r^o_H = 36 nm**	39.5 ± 1.5	40.7 ± 0.3	0.15	0.12	-7.1 ± 1.0	N/A
**AuNPs r^o_H = 58 nm**	60.7 ± 0.2	63.0 ± 1.3	0.05	0.02	-7.7 ± 0.1	N/A
**PS r^o_H = 110 nm**	114 ± 1	113 ± 1	0.13	0.02	N/A	N/A
**PS r^o_H = 30 nm**	32.9 ± 0.1	34.6 ± 0.4	0.02	0.02	N/A	N/A

* Hydrodynamic radius calculated using cumulant analysis as shown in Fig. 1D.

Supplementary Table 2. Conductivity, salt concentration and electrostatic screening length for soft NPs

**Medium**	**Conductivity** (mS/cm)	**[NaCl]** (M)	κ_Q^{-1} (nm)
MilliQ® water	0.001	8.0 x 10^{-6}	107.9
$r_H^0 = 92.5\,nm$	0.025	2.0 x 10^{-4}	21.6
$r_H^0 = 70\,nm$	0.01	8.0 x 10^{-5}	34.1
$r_H^0 = 50\,nm$	0.008	6.4 x 10^{-6}	38.2
$r_H^0 = 45\,nm$	0.013	1.0 x 10^{-4}	29.9
$r_H^0 = 40\,nm$	0.017	1.4 x 10^{-4}	26.2
$r_H^0 = 25\,nm$	0.027	2.2 x 10^{-4}	20.8

Supplementary Table 3. Structural parameters obtained from Supplementary Equation (5) and DDM measurements

C_{ag} **w/w%**	**Mesh size** l **(nm)**
1	116
0.5	220
0.25	311
0.1	491
0.05	695

Supplementary Methods

Rheological measurements

The rheological properties of the agarose hydrogels were measured under oscillatory shear using ARES-G2 rheometer (TA Instruments, U.S.A.). An appropriate amount of agarose was completely dissolved in 100°C MilliQ water. The sample was then injected and sandwiched between parallel plates equipped with a Peltier device for temperature control. The temperature was set at 22°C and evaporation was minimized using a solvent trap provided by the manufacturer. Gelation dynamics were monitored by measuring the storage and loss moduli over time at an angular frequency of 10 rad s^{-1} and strain amplitudes of 1, 2, 50 and 50 % for 1, 0.5, 0.1 and 0.05 % agarose samples, respectively. The strain amplitudes were verified to be at the linear viscoelastic regime. When the

Osmotic pressure in Ficoll solution

Osmotic pressure of Ficoll suspension (0, 50, 100 and 200 mg/mL) was measured with freezing depression point osmometer (μOsmette, Precision Systems). Measurements were performed 6 times consecutively with multiple 50 μL aliquots.

Osmotic pressure of agarose was determined by incubating agarose gels (1% w/v) in Ficoll solutions with various concentration / osmotic pressure. Agarose gels were covered with a dialysis membrane of 15 kDa molecular weight to create the pressure on the gels. Agarose was periodically weighted on an analytical balance to screen water gain / loss. The osmotic pressure was determined at the Ficoll concentration at which the volume of agarose does not change, hence the applied external osmotic pressure Π_{ext}, is in equilibrium with the internal osmotic pressure of the agarose.

mechanical properties were at the steady state, a frequency sweep test was performed at the appropriate strains for each sample, following by a strain sweep test at 10 rad s^{-1} frequency.

Structure factor of soft NPs suspension at ϕ = 0.03%

The structure factor of microgels was measured using the DeGennes narrowing method. Diffusion was measured with DDM and DLS at different q values and normalized with D_0 in the dilute regime ($\phi \rightarrow 0$), so that $\tau_R(q,\phi)/\tau_R(q,\phi \rightarrow 0)$ ~ $D_0/D(q)$ ~ $S(q)$ allow to access for the structure factor $S(q)$. Using microgels r_H^0 = 92 nm, we get $S(q) \approx 1$ which is invariant over the studied q range.

DLS data were obtained with an ALV instrument (CGS-3 goniometer and LSE-5004 correlator) as a function of the scattering angle, using a 633 nm laser, to confirm that the increase in diffusion coefficients (interpreted as particle shrinkage in Fig. 2) is not due to a structural effect. Experiments were performed for soft microgels with r^0_{H} = 92 nm as a function of the concentration (ϕ = [0.009, 3.26%]). The intermediate scattering functions were extracted from measurements of the intensity autocorrelation function[8] at fourteen angles between 20° and 150°. The ISFs were fitted assuming a generalised exponential $f(q,\tau) = e^{-\left(\frac{\tau}{\tau_R}\right)^{\beta}}$. The exponent β was found to be close to 1.

Volume fraction determination of NP suspensions

PS (hard NPs) were compared to microgels (soft NPs) at similar volume fractions. Because microgels can swell or shrink, calculations of ϕ was different for microgels and hard NPs. For hard NPs, we used the following equation to describe ϕ:

$$\phi_{Hard\ NPs} = \frac{w_i \rho_{solv}}{w_i \rho_{solv} + (1-w_i)\rho_{NP}} \qquad (1)$$

And for microgels, we used the approximation provided by Scotti *et al.* as it is valid for low solid volume fractions [1] :

$$\phi_{\mu Gel} = w_i \frac{\rho_{solv}}{\rho_{\mu Gel}} \left(\frac{R_{swollen}}{R_{collapsed}}\right)^3, \qquad (2)$$

Where w_i is the mass fraction of the NP, ρ_{solv} is the density of the solvent, ρ_{NP} or $\rho_{\mu Gel}$ is the density of microgels. For microgels, the term $\left(\frac{R_{swollen}}{R_{collapsed}}\right)^3$ is added to consider the swelling of microgels. $R_{swollen}$ is the radius of the swollen microgel and $R_{collapsed}$ is the radius of the microgel shrunk with temperature as measured by DLS.

Supplementary Note 1 - Examples of DDM measurements in agarose gel.

Intermediate scattering function (ISF), $f(q,\tau)$, shows the dynamics of the particles and can be extracted from the DDM autocorrelation functions, $g(q,\tau)$, as expressed in Eq.1. Extracting $f(q,\tau)$ and applying corrections for sometimes a slow thermal drift in the gel using Eq.11 yields the ISFs reported in Supplementary Fig. 1. All the observed dynamics fits the generalized exponential equation $f(q,\tau) = e^{-(\tau/\tau_R)^\beta}$ with the stretched exponent β close to 1 in water, which indicates NPs are not interacting with each other or the media and polydispersity is not significant, and $0.7 < \beta < 1$ in agarose solutions and gels (with few exceptions of $\beta \approx$ 0.5-0.6), highlighting particle-gel interactions depending on the NPs type and size (see Supplementary Fig. 4).

After filling a capillary with agarose gel, we noticed a slow gelation process affecting the particle dynamics. To avoid the effect of slow relaxation, we performed DDM experiments as a function of the waiting time, ranging from 15 minutes to 3 days, with t=0 the time at which the capillary was filled with a solution. We found that a resting time of 16h was necessary prior to any DDM measurement in order to reach data at the steady state in the agarose gel. This is expressed in Supplementary Fig. 2 (B, D, F and H), where the diffusion coefficient of hard NPs is independent of time after a relaxation period of 16h. We also show the relaxation time τ_R approximately follows q^{-2}. Similar results were found for soft NPs as well (Supplementary Fig. 3).

Supplementary Note 2 - Rheological measurements

Agarose was found to display an apparent Newtonian behavior at very low concentrations (0.05%) as shown by the power-law increase in the loss modulus, G'', with frequency in Supplementary Fig. 5. Above this concentration, agarose demonstrates a non-Newtonian behavior at the studied frequency range because of interchain interactions. An expected sol-gel transition occurs close to C_{ag} = 0.1% characterized by the storage and loss moduli following a power-law scaling of the form $G' \sim G'' \sim \omega^n$, where n is a critical relaxation exponent. The sol-gel transition occurs at an agarose

concentration between 0.1 and 0.5% evidenced by the independency of *G'* to ω. The storage moduli of these gels are 330 ± 10 kPa and 1890 ± 220 kPa for C_{ag} = 0.5% and 1% agarose, respectively.

Supplementary Note 3 - Effect of the osmotic pressure on microgel diffusion

We investigated the soft NP shrinkage as a function of osmotic pressure by adding Ficoll 400 kDa to solution of NP microgels and evaluate the minimum pressure required to shrink the microgels to half their swollen size, as it was observed in agarose (Figure 2).

We measured the diffusion coefficients of soft and hard NPs using DDM and the osmotic pressure as a function of Ficoll concentration (see Supplementary Fig. 6A & C). The mathematical relation between the osmotic pressure and Ficoll concentration (red curve in Supplementary Fig. 6A) was obtained by a second order polynomial fit (expected for a virial expansion). Microgels and PS NPs of about the same size (r^0_H = 92.5 and 110 nm) were used at $\phi_{microgel}$ = 0.0089% and ϕ_{PS} = 0.0014% in Ficoll solutions of 10, 20, 30, 40, 50 and 75 mg/mL in Supplementary Fig. 6C.

As Ficoll concentration increases both osmotic pressure and viscosity increase. However, we found that the ratio $\frac{D_{PS}(\phi_{Ficoll})}{D_{\mu Gel}(\phi_{Ficoll})} = \frac{r_{H_{\mu Gel}}(\phi_{Ficoll})}{r_{H_{PS}}}$ decreases as the Ficoll concentration is increased (inset of Supplementary Fig. 6C) suggesting that the microgel particle shrinks, *i.e.* $r_{H_{\mu Gel}}(\phi_{Ficoll})$ decreases. The viscosity of the solution can be estimated by using the Stokes-Einstein relation and considering the hard PS particle does not change size so that

$$\eta(\phi_{Ficoll}) = \frac{D_{PS}(\phi_{Ficoll}=0)}{D_{PS}(\phi_{Ficoll})\,\eta(\phi_{Ficoll}=0)} \qquad (3)$$

This allows extraction of the microgel radius r_H as a function of Ficoll concentration from solely measured quantities using the following expression

$$r_H(\phi_{Ficoll}) = r_H^0 \frac{D_{\mu Gel(H_2O)}}{D_{\mu Gel(\phi_{Ficoll})}} \frac{D_{PS(\phi_{Ficoll})}}{D_{PS(H_2O)}} \qquad (4)$$

Evolution of r_H as a function of the measured osmotic pressure confirmed that particles are shrinking as the osmotic pressure is increased. We found that the smallest size attainable by the microgel ($r^0_H = 92.5$ nm) in Ficoll is $r_H \approx 60$ nm, which is obtained at a concentration of 50 mg mL^{-1} Ficoll. This concentration yields an osmotic pressure $\Pi_{osm} = 9.1$ kPa (see Supplementary Fig. 6A) which shrinks the microgels similarly to other stimuli such as temperature and concentration (Supplementary Fig. 6D). The measured thermal shrinking observed in this study is consistent with other reports[2, 3].

The osmotic pressure of agarose was calculated from its swelling / deswelling in Ficoll solutions. The concentration of Ficoll at which the agarose gels ($C_{ag} = 1\%$ w/w) does not swell / deswell ($\Delta V = 0$) was estimated by a linear fit (Supplementary Fig. 6B). At this point, the internal osmotic pressure, including the elastic and mixing osmotic pressures, is equivalent to the external osmotic pressure. Therefore, the agarose gel ($C_{ag} = 1\%$ w/w) has an osmotic pressure of $\Pi_{osm} = 89$ Pa equivalent to 3.1 mg mL^{-1} Ficoll, which is too weak to induce any microgel shrinkage.

Supplementary Note 4 - Zeta potential of hard and soft NPs

Zeta potential was assessed by measuring the electrophoretic mobility of the nanoparticles using DLS for NPs in 4 mM NaCl solutions. Values are presented in Supplementary Table 1 for a range of size for both hard AuNPs and soft microgels at two different temperatures.

Supplementary Note 5 - Dynamics in saline solutions

We investigated the effect of adding salt on the diffusion of the NPs in agarose gel at $C_{ag} = 0.5$ % w/w. A larger mesh size of the gel is expected with higher ionic strength solutions [4]. Generally, those ions contribute in reducing water-polymer interactions consequently promoting polymer-polymer interactions and leading to larger chain aggregates as well as pores [5, 6]. With bigger pores, we expect no overlap of the EDL and thus no microgel shrinking. Supplementary Fig. 7 shows the DDM-measured diffusion coefficient D_G of hard Au NPs (two sizes) and soft microgel particle (one

size). With salt addition, we found D_G values of the soft particles agree well with the prediction of the Kang et al. model for the hard particles suggesting that the soft microgel particle do not shrink. Using Eq. 4 to calculate the size r_H of microgels, we obtained a size that corresponds to the fully swollen microgels r^0_H observed in the diluted regime.

Supplementary Note 6 - Calculations of the agarose mesh size and interaction distance H

The mesh size l was determined based on the first hard immobilized particle in agarose gels. Because softer gels are not dense enough to trap NPs of 220 nm in diameter, we used the expression

$$l = L\sqrt{C^*{}_{ag}/C_{ag}} \tag{5}$$

where L is the length of the fiber, C^*_{ag} the overlap concentration of agarose and C_{ag} the concentration of agarose. This approach allows to estimate the mesh size l for $C_{ag} \leq 0.25\%$ w/w using the mesh size of agarose 0.5% as a reference to evaluate $C^*_{ag} = 0.097\%$ w/w. Calculated values of l in Table S3 are in good agreement with previously published values.[7]

In agarose gels, the fiber-particle distance H is obtained using the following expression:

$$H_{Agarose} = \frac{l}{2} - r_H^0 \tag{6}$$

Supplementary Note 7 - Elastic energy of a spherical particle

The elastic energy U of a soft spherical particle of elastic modulus E is described by its elastic potential energy per unit volume $U_v = 1/2E\xi^2$ with ξ being the strain on the particle so that we have

$$dU = \frac{E}{2}\xi^2 dV \tag{7}$$

and the strain

$$\xi = \left(\frac{R_0 - r}{R_0}\right), \tag{8}$$

where R_0 is the initial radius of the particle and r is the shrinked layer of the particle. The potential energy required to compress a soft sphere to a fraction α of its original size (α being the shrinkage ratio of the particle) is given by:

$$U = \frac{E\pi {R_0}^3}{15}(6\alpha^5 - 15\alpha^4 + 10\alpha^3 - 1). \quad (9)$$

Therefore, for a particle of a weak elastic modulus (E = 1 kPa), initial radius of R_0 = 50 nm, and α = 2/3, we get $U/k_B T$ = 1.34. The required energy for shrinking by α this type of particle then needs only 10-20 agarose fibers within its vicinity, which is reasonable considering the fractal nature of agarose and the non-homogenous fiber distribution in its matrix.

Supplementary References

1. Scotti A, *et al.* The role of ions in the self-healing behavior of soft particle suspensions. *Proc. Natl. Acad. Sci. U.S.A.* **113**, 5576-5581 (2016).

2. Fernández-Nieves A, Fernández-Barbero A, Vincent B, Nieves FJdl. Osmotic de-swelling of ionic microgel particles. *J. Chem. Phys.* **119**, 10383-10388 (2003).

3. Sierra-Martin B, Frederick JA, Laporte Y, Markou G, Lietor-Santos JJ, Fernandez-Nieves A. Determination of the bulk modulus of microgel particles. *Colloid. Polym. Sci.* **289**, 721-728 (2011).

4. Waki S, Harvey JD, Bellamy AR. Study of agarose gels by electron microscopy of freeze□fractured surfaces. *Biopolymers* **21**, 1909-1926 (1982).

5. Ioannidis N, Bowen J, Pacek A, Zhang Z. Manufacturing of agarose-based chromatographic adsorbents – Effect of ionic strength and cooling conditions on particle structure and mechanical strength. *J. Colloid Interface Sci.* **367**, 153-160 (2012).

6. Park TG, Hoffman AS. Sodium chloride-induced phase transition in nonionic poly(N-isopropylacrylamide) gel. *Macromolecules* **26**, 5045-5048 (1993).

7. Jiang L, Granick S. Real-Space, in Situ Maps of Hydrogel Pores. *ACS Nano* **11**, 204-212 (2017).

8. Martinez VA, Thijssen JHJ, Zontone F, Megen Wv, Bryant G. Dynamics of hard sphere suspensions using dynamic light scattering and X-ray photon correlation spectroscopy: Dynamics and scaling of the intermediate scattering function. *J. Chem. Phys.* **134**, 054505 (2011).
